# Telerehabilitation to improve outcomes for people with stroke: study protocol for a randomised controlled trial

**DOI:** 10.1186/1745-6215-13-233

**Published:** 2012-12-05

**Authors:** Nicola Saywell, Alain C Vandal, Paul Brown, H Carl Hanger, Leigh Hale, Suzie Mudge, Stephan Milosavljevic, Valery Feigin, Denise Taylor

**Affiliations:** 1 Health and Rehabilitation Research Institute, AUT University, Private Bag 92006, Auckland, 1142, New Zealand; 2 Centre for Research, Knowledge and Information Management, Counties Manukau District Health Board, Auckland, New Zealand; 3 Older Persons Health Specialist Services, Canterbury District Health Board, Christchurch, 8140, New Zealand; 4 Centre for Physiotherapy Research, University of Otago, Dunedin, 9054, New Zealand; 5 Health Sciences Research Institute, University of California, Merced, CA, 95343, USA; 6 National Institute for Stroke and Applied Neurosciences, AUT University, Auckland, New Zealand

**Keywords:** Stroke, Telerehabilitation, Transition, Resources, Cost-effective, Exercise, Physiotherapy

## Abstract

**Background:**

In New Zealand, around 45,000 people live with stroke and many studies have reported that benefits gained during initial rehabilitation are not sustained. Evidence indicates that participation in physical interventions can prevent the functional decline that frequently occurs after discharge from acute care facilities. However, on-going stroke services provision following discharge from acute care is often related to non-medical factors such as availability of resources and geographical location. Currently most people receive no treatment beyond three months post stroke. The study aims to determine if the Augmented Community Telerehabilitation Intervention (ACTIV) results in better physical function for people with stroke than usual care, as measured by the Stroke Impact Scale, physical subcomponent.

**Methods/design:**

This study will use a multi-site, two-arm, assessor blinded, parallel randomised controlled trial design. People will be eligible if they have had their first ever stroke, are over 20 and have some physical impairment in either arm or leg, or both. Following discharge from formal physiotherapy services (inpatient, outpatient or community), participants will be randomised into ACTIV or usual care. ACTIV uses readily available technology, telephone and mobile phones, combined with face-to-face visits from a physiotherapist over a six-month period, to help people with stroke resume activities they enjoyed before the stroke. The impact of stroke on physical function and quality of life will be assessed, measures of cost will be collected and a discrete choice survey will be used to measure preferences for rehabilitation options. These outcomes will be collected at baseline, six months and 12 months. In-depth interviews will be used to explore the experiences of people participating in the intervention arm of the study.

**Discussion:**

The lack of on-going rehabilitation for people with stroke diminishes the chance of their best possible outcome and may contribute to a functional decline following discharge from formal rehabilitation. Best practice guidelines recommend a prolonged period of rehabilitation, however this is expensive and therefore not undertaken in most publicly funded centres. An effective, cost-effective, and preference-sensitive therapy using basic technology to assist programme delivery may improve patient autonomy as they leave formal rehabilitation and return home.

**Trial registration:**

ACTRN12612000464864

## Background

Stroke is the leading cause of disability in ageing populations of developed countries [1]. In New Zealand around 45,000 people live with stroke and only 30% are independent in activities of daily living [2,3]. International figures indicate that only 40% of people with stroke achieve even minimal levels of physical activity [4]. A low level of physical activity over time leads to a gradual physical deterioration and loss of function, which has a detrimental impact on participation and quality of life (QoL) [5,6]. People with stroke are more likely to have a significantly reduced QoL than an age-matched population [7], to the extent that in an Australian study, 8% of people with stroke rated their quality of life as equal to or worse than death [8]. A recent meta-analysis of physiotherapy interventions in chronic stroke pooled the results from 15 trials of community-based physiotherapy and reported a favourable effect on mobility-based measures; a meta-analysis of primary mobility-based outcomes showed a significant effect of the intervention (effect size 0.29, 95% CI 0.14, 0.45) [9]. Participants with stroke in a qualitative study found telerehabilitation to have a broader range of benefits than anticipated by the researchers. Participants expressed perceived increased autonomy and appreciated the flexible and self-directed nature of telerehabilitation. The therapist’s role changed over time from a directive approach to a more motivational and facilitatory one [10]. There is evidence that even low intensity activity-based interventions improve independence in community-dwelling adults who have had a stroke and can positively influence QoL [11-13]. It appears that ongoing participation in physical activity prevents the decline in functional independence commonly seen in people with stroke once their rehabilitation has ceased. National and international stroke best-practice guidelines reflect this, and recommend long term engagement in some form of physical activity after discharge from hospital [14-16]. A Cochrane review showed that people with stroke who received long-term rehabilitation (up to one year post-stroke) had increased odds of a positive outcome and were more likely to be independent in functional tasks [11]. Despite this, in New Zealand many people with stroke do not receive any physiotherapy beyond three months post-stroke [17]. The benefits gained during initial rehabilitation are often not sustained in the long term, as people reduce activity levels and cease engagement in exercise programmes, leading to functional deterioration [18]. A consequence of this functional deterioration is high rates of admission to hospital and residential care within the first 12 months post-discharge [19]. Healthcare resources are limited, and whether an individual receives appropriate and on-going stroke services is often related to non-stroke factors, such as resource availability, geographical location, age, and personal finances [20,21]. Patients are frequently told they are discharged from therapy because they have reached a plateau, when the decision to discharge is likely to be more complex and includes consideration of resource availability [9,22]. People with stroke are often disappointed when they are discharged from rehabilitation; their disappointment is related to unmet expectations of recovery and a desire to receive rehabilitation until they perceive it is no longer needed [21,23]. Although the New Zealand Best Practice Guidelines recommend long-term rehabilitation, there are questions about feasibility [24] and the cost of ongoing rehabilitation. Our aim is to deliver an intervention that increases self-directed activity with a return of the locus of control to the person with stroke, utilising telerehabilitation to reduce the cost of rehabilitation. Technologies available for telerehabilitation delivery range from telephones and mobile phones, which are available in the majority of homes, through to more expensive internet-based solutions, virtual reality and robotic systems. In a review of home-based physical activity interventions for older adults, four of seven studies used telephone communication to encourage continued participation, with encouraging results. There were high levels of participation over the short and long term (86% to 93% and 44% to 68% of people adhering to their exercise programme respectively) [25]. It has been suggested that physiotherapists are ideally placed to assist people with stroke transition from hospital to home [26] but there has been considerable international professional resistance to using a telerehabilitation-based service, with a view that the loss of hands-on treatment may lead to poorer outcomes [27]. However, evidence from studies in various chronic health conditions show that telerehabilitation approaches can be successful in improving outcomes [28-30]. The American Physical Therapy Association is revising its stance to support telerehabilitation as a delivery method [31]. One feasible approach to telerehabilitation in New Zealand may be to provide limited home visits by a physiotherapist, a structure that has been used successfully by the Otago Exercise Programme (OEP) for falls prevention [32]. The OEP programme provided a limited number of home visits supplemented by telephone calls [33,34]. For ACTIV to be adopted by Regional Health Services, there must be clear evidence that it produces a significant improvement to people’s outcomes after stroke in a cost-effective manner. Telerehabilitation is a relatively new mode of delivery of interventions [35], with limited evidence concerning the net costs, the effectiveness and the cost-effectiveness compared with standard care. The current study will be among the first to assess the benefits and costs of an intervention that uses telerehabilitation as part of the package.

## Methods/design

### Study aims

The overarching aim of this study is to investigate ACTIV for people with stroke to improve the transition from hospital to community living. The specific aims are to:

1. Determine the effect of ACTIV on physical function

Primary research question: At the end of the 6-month intervention, will ACTIV improve physical function in people with stroke compared to a usual care control group, as measured by the physical subcomponent of the Stroke Impact Scale (SIS 3.0)?

Secondary research question 1: At the 12-month follow-up, will any physical gains achieved by the intervention group at the end of the intervention be maintained as measured by the physical subcomponent of the SIS 3.0?

Secondary research question 2: At the end of the 6-month intervention and at follow-up, will ACTIV improve physical function in people with stroke, compared to a usual care control group as measured by the physical performance measures and the stroke self-efficacy questionnaire?

2. Determine the effect of ACTIV on health outcomes and quality of life.

Secondary research question 3: Over the study period, will ACTIV improve health outcomes and quality of life in people with stroke compared to a usual care control group as measured by the SIS 3.0?

3. Determine the effect of ACTIV on hospital and residential care admission rates.

Secondary research question 4: Over the study period, will participation in ACTIV result in a reduction in hospital admissions compared to the usual care control group?

4. Determine the cost and cost-effectiveness of ACTIV.

Secondary research question 5: Over the study period, will ACTIV be cost-effective compared to usual care in reducing aspects of the burden of care in people with stroke?

5. Explore participant experience of and satisfaction with ACTIV.

Qualitative question: What are the experiences, in particular, the perceived benefits and challenges for participants in ACTIV?

### Study design

**Figure 1:**
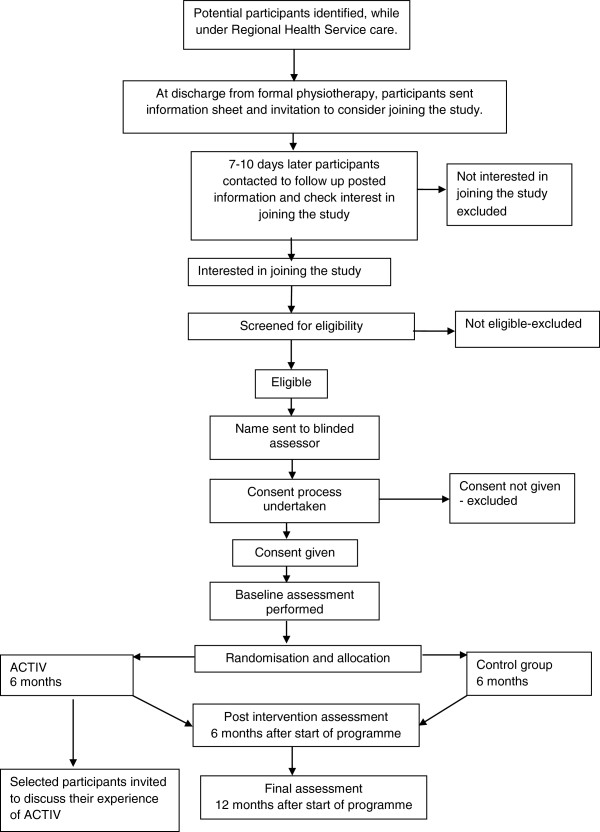
Design and procedure of study.

This is a multi-site, two-arm, assessor blinded, parallel randomised controlled trial (RCT). Alongside the RCT, an economic evaluation will compare the cost-effectiveness of the intervention with usual care and preferences for stroke rehabilitation delivery, in addition, a qualitative component will explore the experiences of participants with the intervention. Enrolment will occur in New Zealand in the North Island at Middlemore Hospital (South Auckland), North Shore Hospital (North Auckland), and in the South Island at The Princess Margaret Hospital (Christchurch), and Dunedin Hospital (Dunedin). People will be eligible for inclusion in the study at the time of discharge from standard physiotherapy. Standard physiotherapy will include inpatient rehabilitation in general medical or specialist stroke care facilities, and outpatient or community physiotherapy post-discharge from hospital. Outcome assessments will be conducted at baseline, immediately after the six-month intervention and at six months post-intervention by an assessor blinded to group allocation. Figure 1 outlines the study design and procedure.

### Ethical approval

The New Zealand Multi-regional Ethics Committee gave ethical approval for this study (MEC 11/11/089) and each of the centres where recruitment is to take place approved the study. Māori consultation was undertaken in each centre to ensure cultural values that may differ between *Iwi* (tribe) were given appropriate consideration. All participants will give written informed consent prior to participating in the study.

### Study sample

#### Sample size

Sample size computations are based on a 5% per comparison significance level and a two-tailed critical region. Due to the variation in response to physiotherapy intervention after stroke a conservative approach has been taken with the assumption that there will be no correlation between baseline measures and final outcome. Two previous studies [36,37] have indicated consistent baseline SDs for the physical function subcomponent of the SIS, for a pooled baseline SD of approximately 21.7. A naïve combination of the clinically important differences (CID) from Lin and colleagues in 2010 [36], based on a geometric mean of the elements, yields a CID for the SIS physical function subcomponent score of 5.3, corresponding to an effect size of about 0.25. In their rehabilitation study, Marsden and colleagues 2010 [37] obtained standard errors for the mean differences in measurement, of 9.5 and 6.6 for their control and treatment groups, respectively. Using these values, 38.4 participants per arm will be required to detect the target effect size with 80% power, based on the appropriate *t*-distribution for the difference of two such changes from baseline. A total sample size of 96 will be required, allowing for an attrition rate of 20% over the 12-month research period. With these numbers, the probability is approximately 80% that the study will detect a treatment difference, if the true difference between the changes in ACTIV and usual care control group is at least 5.4 points in the physical subcomponent of the SIS3.0.

#### Recruitment

Participants will be recruited via hospital-based stroke and rehabilitation services. In all four hospitals, potential participants will be identified via the Community-Based Rehabilitation Team or Community Stroke Rehabilitation Team. In addition, discharges from acute medical wards and rehabilitation wards will be checked for people discharged directly from these services. To access people with stroke who are under 65 years of age, in the Christchurch area, discharges from Burwood Brain Injury Service will be checked. Eligible participants will be mailed an information sheet and letter of introduction to the project; this will be followed up with a telephone call to ascertain interest. Potential participants who identify as Māori or who express a need for more information will be given the opportunity for a face-to-face discussion of the information sheet. For those interested in participation, telephone screening will be undertaken with the potential participant or their spouse/carer once they have been discharged from hospital care, to establish eligibility. If they meet selection criteria an assessor will make an appointment for the baseline assessment to be carried out. All assessments will take place in the participant’s home.

### Inclusion criteria

People will be eligible for inclusion if they have had a first ever hemispheric stroke of haemorrhagic or ischaemic origin; are over the age of 20 years; have been discharged from inpatient, outpatient and community physiotherapy services to live in their own home (participants involved in other forms of therapy such as Occupational Therapy, Tai Chi or community exercise programmes will not be excluded); have medical clearance from their General Practitioner to participate in a low to moderate level activity programme; score at least 3 on a telephone cognitive screening questionnaire [38]; have a limitation in physical function of leg, arm or both.

Limitation in physical function of the leg will be established if patients have a score between 4 and 6 on the Functional Ambulation Category (FAC) [39] and in the case where they score 6, also answer ‘No’ to at least one of the two walking parameter questions below:

1. Can you get across the road at the traffic lights, in the time the green man is showing?

2. Are you able to walk 400m? (that is, ¼ mile, one to two blocks)

Limitation in physical function of the arm will be established using the questions below, if they answer ‘Yes’ to at least one question in section A and ‘No’ to at least one question in section B.

A. With your affected arm are you able to:

1. Switch on a light?

2. Bring a glass of water to your mouth?

3. Move your fingers and thumb independently?

B. Are you able to:

1. Use a keyboard equally with both hands?

2. Holding a pencil with your affected hand make rapid dots on a piece of paper?

3. Take a spoonful of liquid to your mouth without spilling it or bending your neck?

### Exclusion criteria

People will be excluded if they have a confirmed brain stem or cerebellar stroke or are unable to understand and speak basic-level English. Involvement in ACTIV requires frequent communication with a physiotherapist, in person, by telephone and by text message (SMS message), so using an interpreter is considered impractical.

### Randomisation

Randomisation will occur after the baseline assessment and participants will be assigned to one of two groups, ACTIV or usual care control. Randomisation will be stratified by centre (four locations) with recruitment targets of 32 participants from each of the North Island centres and 16 from each of the South Island centres, commensurate with the number of stroke admissions expected in each location. Stratified block randomisation will be used according to geographic centre and baseline mobility. Baseline mobility will be decided by the FAC: either ‘less mobile’ if they have a level of five or below or ‘more mobile’ if they have a level of six. Given the small numbers, random block sizes will be used according to a plan that ensures, according to simulations, a probability smaller than 0.1% that balance will be broken across strata by four participants or more. The randomisation software will be coded and tested by the study statistician, then handed over to a third independent party for random number generator seeding, execution of allocation, and day-to-day management of the randomisation. The recruiters, assessors and personnel involved in data management and analysis will be blinded to treatment assignment.

### Intervention - ACTIV

The Augmented Community Telerehabilitation Intervention (ACTIV) is a 6-month standardised programme delivered in the participant’s home, focusing on two functional categories: ‘staying upright’ and ‘using your arm’. Within each functional category there are standard exercises addressing key components of the defined function. For each component there are parameters to select and modify, allowing the programme to be individualised. The parameters that can be changed include the support received during an exercise, the number of exercises prescribed and the environment in which the exercise will take place. ACTIV will be delivered by a physiotherapist who has completed a training package that provides a) information on the rationale of ACTIV, b) a menu of exercises with guidelines for treatment selection, c) information on goal setting and d) information to facilitate patients in the transition to increased independence. Patient-centred goals will be set at the first home visit (assessment visit) and the physiotherapist will then select exercises and activities aimed at incremental attainment of these goals. The physiotherapists will have telephone and internet access to a consultant physiotherapist with expertise in this programme and in stroke rehabilitation, with who they can discuss any concerns/questions regarding provision of the programme.

ACTIV consists of four home visits (weeks 1, 2, 12 and 25), five telephone calls (weeks 1, 4, 8, 16 and 20) and text messaging (twice weekly for the first 10 weeks and once weekly for the following 16 weeks). Some people with stroke re-access services following discharge from formal therapy. No effort will be made to prevent the intervention group receiving additional care either publicly or privately.

### Initial assessment and exercise prescription

The physiotherapist will work with the participant to identify a goal using the phrase ‘What do you want to do most?’ Once this activity has been selected, the therapist will then explore the participant’s perception of why they are unable to complete this activity and will then observe them attempting the activity, noting the aspects they are unable to complete. If the desired activity is a significant distance from their current ability the therapist will work with the patient to establish interim steps that will act as a bridge between their current ability and their goal. The evaluation of the observed activity will allow the therapist to select appropriate exercises and level of difficulty. Exercises will be selected from a standard menu (see Additional file 1: Appendix 1 for the lower limb menu) and modified by mutual agreement between participant and therapist, to pursue and attain the desired activity. This will result in an individualised programme based on the assessment of the participant’s goals and ability. One sheet for each selected exercise, giving clear detail of parameters and a diagram of the exercise, will be left with the participant in a manual (see Additional file 2: Appendix 2 for an example of one exercise).

### Follow-up visits

The physiotherapist will check that prescribed exercises are being performed effectively and safely. If the participant is able to perform the prescribed exercises with ease, some parameters will be changed to maintain the challenge of the exercise. If the selected goal has been reached the participant will be encouraged to consider another desired activity.

Information will also be collected about exercise completion and facilitators and barriers to exercise. This will help personalise subsequent communication by phone and text messaging. The participant will be encouraged incrementally to take control over deciding on a desired activity and selecting exercises that help them reach that activity, to reduce reliance on the physiotherapist over the 6-month treatment phase.

### Telephone calls

A structured telephone interview will be used to check on progress, satisfaction with the programme and the occurrence of any barriers to completion of the exercises. Information from each interview will be used to help formulate a strategy to maximise participation in the programme. For example, if a participant reports that they are unable to complete the exercises because they do not understand the instructions, the physiotherapist will clarify or change the exercise instructions or parameters. If a participant reports that they are finding the programme too easy, the physiotherapist can provide modifications to increase the difficulty level. No new exercises will be prescribed over the phone.

### Text messages

Encouragement to continue with the exercise programme will be provided by the physiotherapist. This encouragement will be via brief text messages acknowledging progress so far and focussing on goal attainment. Participants will be encouraged to use mobile phones provided if possible, but if that is not possible, e-mail messages will be sent as an alternative. Modifications of the exercises already prescribed will be suggested via text message but no new exercises will be given. Simple checks on progress will be undertaken (with those able to use text messaging to reply).

### Telecommunication technology

Mobile phone ownership and acceptability of use has been investigated in New Zealand through focus groups undertaken by the authors in groups of community dwelling people with stroke. Preliminary results showed approximately 60% of people owned and used a mobile phone, with others having access to one if needed. However, it is acknowledged that some people will not have access to a mobile phone, so in order to increase the opportunity to participate in this trial, we will provide a mobile phone to all those undertaking ACTIV, who do not own a phone. Participants who do not own a phone will be instructed on the use of a mobile phone and then encouraged to use it to access the text messages sent as part of ACTIV. We trialled a variety of low-cost phones and selected the most straightforward devices with large keys and easy-to-read screens. Plain English instructions will be left with all ACTIV participants to improve their ability to use the mobile. A small credit will be put on all ACTIV participants’ phones, to ensure that cost of messaging is not a barrier to continuation of the programme.

### Additional support

If the participant requests information about stroke-related issues beyond the exercise programme, the physiotherapist will have information sheets commonly in use, which will be provided by the Stroke Foundation (a not-for-profit organisation supporting and representing people with stroke in the community) and will be kept in the folder used by the physiotherapist for assessment forms and other paperwork. These can be provided as required. The participant can contact the physiotherapist at any stage if an adverse event occurs, if there is confusion about the programme and instruction, or if they are unable to perform the exercises.

### Contingency planning

In the event of a concern about the participant’s health or wellbeing, the physiotherapist may gain consent from the participant to contact their emergency contact person. As a registered health care professional, the physiotherapist will access appropriate medical assistance, as they would in any other clinical setting.

### Discharge visit

The physiotherapist will check on goal achievement. If the goal has been reached the participant will be encouraged to consider the next thing they would like to achieve. If the original goal has not yet been reached the physiotherapist will encourage the participant to note the steps that have been achieved and plan the next steps needed. Encouragement will be given to continue with the exercise programme independently, focussing on exercises in ACTIV that have been useful.

### Usual-care control group

Some people with stroke re-access services following discharge from formal therapy; no effort will be made to prevent the control group receiving additional care either publicly or privately. However, participation in physical activity will be ascertained during a monthly phone call by a research assistant, independent of the study and blinded to group allocation. Participants in the control group will undergo the three assessments from the blinded assessor at baseline, 6 and 12 months.

### Data monitoring

A Data Monitoring Committee (DMC) will monitor the progress of the study. The DMC consists of the study statistician, and two researchers independent of the study. The Health Research Council of New Zealand, as the funder of this research, reviewed the proposal at its Data Monitoring Core Committee and agreed that as a low-risk study, a wholly independent DMC was not required and the partial unblinding of the study statistician was acceptable.

### Outcome measures

#### Baseline data

Baseline demographic data will be collected from all participants at the initial assessment by the blinded assessor. All quantitative outcome measures will be collected at baseline, immediately after the intervention (approximately six months after baseline assessment) and 6 months after the intervention has finished (approximately 12 months after baseline assessment).

### Primary outcome

The primary outcome measure is the physical function subcomponent of the SIS 3.0. [40]. The SIS 3.0 consists of 59 questions divided into eight domains; strength, hand function, mobility, activities of daily living, emotion, memory, communication and social participation. The four physical domains (strength, hand function, mobility and activities of daily living) can be summed to generate the physical subcomponent score. This is reported as a normalised summary score, has excellent test-retest reliability with interclass correlation (ICC) of 0.98, and the SIS has been shown to be responsive to change in this population [41]. Rasch analysis of the SIS indicates that the items are uni-dimensional with an excellent range of item difficulty [40].

### Secondary outcomes

#### Physical performance measures

Two simple physical performance measures will be used as follows: (1) a Jaymar® hand-held dynamometer (Sammons Preston, Rolyan, Bolingbrook, IL, USA) will be used to ascertain grip strength. The reliability coefficient for grip strength in the paretic arm of people with stroke is > 0.80 [42]; (2) the step test will be used to assess dynamic balance. This has been shown to have good test-retest reliability in stroke with an ICC of 0.88 [43] and has been used in RCTs of stroke-related interventions [44,45].

#### Self-efficacy

The Stroke Self-Efficacy Questionnaire (SSEQ) [46] will be used to collect data relating to the stroke participant’s confidence in their ability to undertake daily tasks that may have been difficult since their stroke. The SSEQ has been shown to have good face validity and feasibility when used in the recovery period following stroke. Its criterion validity was 0.80, when compared with the Falls Efficacy Scale [46]. Self-efficacy has been shown to be an important predictive factor in ongoing physical activity post-stroke [4].

#### Health outcomes and the impact of stroke

The SIS measures changes in both body structure and activity, and will be used to ascertain changes in health outcomes and the impact the stroke has had in various areas of the participants life; all eight domains and the overall stroke recovery rating will be used [36]. Test-retest reliability is excellent (ICC > 0.90) for all but the emotion domain, which has an ICC of 0.68 [47].

#### Hospital and residential care admission rates

Acute or emergency admission to hospital and respite or permanent admission to residential care will be collected from electronic records and stroke participant/carer responses. However only those that are a result of the stroke will be used in the economic evaluation of the intervention.

#### Economic evaluation

This study incorporates an economic evaluation alongside the study. It takes the perspective of the health funder and compares ACTIV with usual care. Direct healthcare cost associated with stroke survivorship will be assessed for all participants. One-year direct cost of stroke will be estimated using hospital electronic records and stroke participants’/carers’ survey responses. Costs will be reported for one year post-stroke and modelled over the lifetime post-stroke. For cost-effectiveness of the intervention, the cost of health services usage obtained from electronic records and the survey responses will be compared between the two groups. Responses to the EuroQol (EQ)-5D questionnaire and willingness to pay for the services will be compared to ascertain the cost utility and cost benefit of the intervention. Although no differences in mortality are expected, differences in utility will be modelled over the expected life span for stroke survivors in the community to identify the long-term cost-effectiveness of the intervention. Probabilistic sensitivity analyses will be performed to assess the robustness of the results to changes in key parameters [48]. A discrete choice experiment will be used to ascertain patient preferences for rehabilitation [49,50].

#### Qualitative component

At the end of the intervention, a purposive sample of participants in the experimental group will be interviewed to include, as far as practicable, a range of sociodemographic factors, including age, ethnicity, education, marital status, and mobility. Semi-structured in-depth interviews will be conducted by one of the researchers in a mutually agreeable location. Topics that will be explored will include: what the intervention meant for the participant, any benefits derived from the intervention, interest in and barriers to continuing the exercises and ideas that would improve the intervention. Of particular interest are participants’ views of whether they were able to take steps towards achieving a desired activity and if successful, whether the skills learnt could be applied to another desirable activity. Interviews will last between 1.0 and 1.5 hours, be audio-recorded and fully transcribed. A constant comparison process will be used for each qualitative component; researchers will reflect on and discuss completed interviews and revise the question schedule accordingly to ensure a broad capture of new important information.

The research questions in the qualitative component of the study are evaluative in nature, emphasising and exploring issues of utility, feasibility and propriety [51]. After the first six participant interviews, theoretical sampling will be used to guide further recruitment and interviewing, to capture the stories of participants with potentially different experiences to challenge emerging themes. Recruitment will cease at data saturation when no new themes emerge from the interviews. It is anticipated that data saturation will be reached after approximately 20 participants have been interviewed. Each physiotherapist will be interviewed about their experience of delivering ACTIV. The interview will include questions about positive and negative aspects of the programme, suggestions for programme modifications and anything that would have assisted in the delivery of the intervention.

#### Adverse events

Every participant in the intervention and the control arm of ACTIV will be telephoned monthly by a research assistant, independent of the study and blinded to group allocation. The research assistant will have a comprehensive understanding of the meaning of adverse events in the context of this study. An adverse event is defined as any untoward medical occurrence in a participant that does not necessarily have a causal relationship with the study treatment [52]. They will note all events and establish whether the participant feels that the event is attributable to the intervention or not. A report of all adverse events will be collated by a senior research officer, who will report to the DMC monthly, to ensure that any significant increase in adverse events in the intervention group as compared with the control group can be assessed. Adverse events will be coded according to the Common Terminology Criteria for Adverse Events version 4.0.

#### Physical activity

During the adverse event phone call each participant in the study will also be asked about their physical activity participation, including involvement in other therapy. Participants will be asked if they are undertaking therapy or organised exercise (apart from this study), and if so, its frequency and duration.

### Data analysis

#### Quantitative analysis

The difference between the two groups in mean changes in the primary outcome between 6 months and baseline will be obtained using analysis of covariance, adjusting for baseline, and accounting for the site using random effects. Secondary analyses will examine all efficacy outcomes over time in a mixed-effects model with centre- and patient-associated random effects. Demographic and other covariates will be assessed for inclusion in the regressions if they differ across treatment arms by more than one pooled SD. They will be retained for adjustment purposes if they reach a significance threshold of 0.1 in the presence of the treatment effect and also in the presence of treatment effect and all such covariates. Trend analyses will be performed by estimating the interaction between treatment arms and piecewise linear functions of the true assessment time. Observations with missing outcome data will be deleted but partial outcome data will be retained in the case of secondary outcomes. Multiple imputation will be used for the production of final results, should covariate values be used for adjustment. Survival analysis of attrition will be used to assess whether differential attrition is extant, and the results used to inform the discussion. Primary analyses will be carried out on the basis of intention to treat. No interim analysis for efficacy will take place.

#### Economic analysis

Resources associated with each stage of the telerehabilitation intervention will be identified by assessing the processes required to implement the procedure and then applying a common unit price to each resource. Incremental cost-effectiveness ratios will be calculated using outcome data and cost data. The net cost will be assessed by comparing the costs for the intervention and control patients. One-way and probabilistic sensitivity analysis will be performed on key parameters to provide estimates of the costs to the Regional Health Services. The results will provide evidence on the net cost of implementing the intervention. Sawtooth software will be used for the experimental design and analysis of the discrete choice experiment.

#### Qualitative analysis

A general inductive approach [53] will be used to analyse qualitative data from both the participant interviews and physiotherapist focus groups. This approach answers specific study research questions by identifying the connections between the research objectives and the summary findings derived from the raw data. It allows findings derived from both the research objectives (deductive) and those arising directly from the analysis of the raw data (inductive) to be identified and associations made to answer specific study research questions. In the analysis process, transcripts will be systematically and thoroughly read by researchers, and a coding framework developed with discussion. As themes emerge on multiple readings, further discussion and adjustments to the coding framework will be made. The main themes will be conceptualised following discussions where agreement will be made on how to collapse the codes into appropriate categories. A researcher not involved with the study will be asked to verify the categorisation of data (consistency check). QSR NVivo 8 software will be used to store the data, record coding and any memos associated with the interviews. Preliminary results of the analysis will be presented to participants for verification (member checks).

#### Adverse events analysis

The occurrence of adverse events will be reported by descriptive statistics only, as we lack sufficient power to test for these events, and strongly expect the number to be low due to the low-risk nature of the intervention.

## Discussion

There are many factors within the health system, including staff availability and financial constraints, which have led to provision of post-stroke rehabilitation for substantially shorter periods than best-practice guidelines recommend. New delivery methods to improve stroke rehabilitation at a sustainable cost are urgently required. Robust evidence of improved outcomes in people with stroke will be required before ACTIV could be considered a useful addition to the current post-stroke clinical pathway. Consensus would be required between therapists, funders and policy makers on the programme’s acceptability, effectiveness and cost-effectiveness. If results of this programme are positive, the proposed model of rehabilitation delivery may act as a framework that can incorporate new technology as it becomes available. A variety of rehabilitation interventions that previously have only been delivered face-to-face have the potential to be delivered remotely.

In the current trial, the choice of four centres from the North and South Island of New Zealand, including rural and urban areas, the range of socioeconomic status, and the wide demographic mix will help to increase the generalisability of the results.

To ensure that after a stroke, people can return to a life that is meaningful to them and resume previous roles, rehabilitation needs to be a lifelong process and should not stop at 3 months or at the end of formal rehabilitation programmes. To provide ongoing rehabilitation in a sustainable way, a low-cost and effective delivery method needs to be found. Using readily available technology will reduce the face-to-face contact and the travel time potentially reducing the cost of intervention. This in turn will allow treatment to continue for longer, which may facilitate the transition from hospital to home and encourage people to direct their own rehabilitation in the longer term. Intervention that combines traditional physiotherapy with telerehabilitation could help people with stroke avoid deterioration in function by improving self-efficacy, facilitating continued improvements in desired activities, thereby reducing the resultant disappointment that so commonly accompanies the patient’s discharge from hospital.

## Trial status

At the time of submission recruitment of participants has just started in all four centres. The results of this study will be submitted for publication in a peer-reviewed journal in a timely manner at the completion of the study, irrespective of the outcomes. The reporting of the trial will adhere to the latest CONSORT statements at the time of manuscript submission.

## Authors’ contributions

NS and DT conceived the study, designed ACTIV, the staff training protocol and drafted the manuscript. AV participated in the study design and planned the statistical analysis of quantitative data. LH gave substantial assistance regarding qualitative analysis. DT and PB designed the discrete choice experiment and PB designed the economic analysis plan. SM and HCH gave considerable assistance with the practicalities of using South Island centres and the liaison required to ensure consistency between sites. SMudge assisted with ACTIV design and staff training protocol, and VF advised on the medical aspects of the protocol. All authors read and approved the final manuscript.

## Authors’ information

NS is a lecturer at AUT University and this project represents her PhD thesis topic. Her particular interest has been the maximisation of function and return to life post-stroke. DT, LH and SMudge are physiotherapists and academics, working and researching the area of neurological rehabilitation. AV is a biostatistician; HCH is a consultant physician and geriatrician with significant interest in stroke rehabilitation. PB is a health economist with an interest in rehabilitation. VF is a neurologist and clinical epidemiologist.

## Supplementary Material

Additional file 1 Menu of exercises - lower limb. Click here for file

Additional file 2 Exercise example. Click here for file
