# Supplementary material for: Telerehabilitation to improve outcomes for people with stroke: study protocol for a randomised controlled trial
Source: Trials. 2012 Dec 5;13:233. doi: 10.1186/1745-6215-13-233 (PMC3543302; doi:10.1186/1745-6215-13-233)
Supplement: Additional file 1 — Menu of exercises - lower limb. [file 1745-6215-13-233-S1.pdf]

Appendix 1 - Menu of exercises - lower limb

Lower Limb Exercise Chart

Participant ID No: ____________________ **Physio Initials:** ____________________________

What they want to do most: ____

____

	**Visit No:**	**Date:**				**Ph Call No:**	**Date:**				**Ph Call No:**	**Date:**			
**Activity**	**Time:**	**Reps:**	**Sets:**	**Speed:**	**BOS:**	**Time**	**Reps**	**Sets:**	**Speed:**	**BOS:**	**Time:**	**Reps:**	**Sets:**	**Speed:**	**BOS:**
**A.** 2 Leg Stand															
**B.** Sit - Stand															
**C.** Stepping Task															
**D.** Step Up															
**E.** Moving Objects															
**F.** Walking															
**G.** Turning															
**H.** Heel Raises															
**I.** Stair Climb															
**J.** 1 Leg Stand															

Examples of altering complexity:

- Eyes closed
- Accuracy
- Holding an object
- Distance
- Change surface

[grey box] Not a recommended parameter, however use if required.
