# Supplementary material for: Telerehabilitation to improve outcomes for people with stroke: study protocol for a randomised controlled trial
Source: Trials. 2012 Dec 5;13:233. doi: 10.1186/1745-6215-13-233 (PMC3543302; doi:10.1186/1745-6215-13-233)
Supplement: Additional file 2 — Exercise example. [file 1745-6215-13-233-S2.pdf]

Appendix 2 - Exercise example

Lower Limb Exercise Sheet

Exercise B	SIT TO STAND

Physio: ______________________________

Phone No: ____________________________

This exercise works on: ____

And helps you improve your: ____

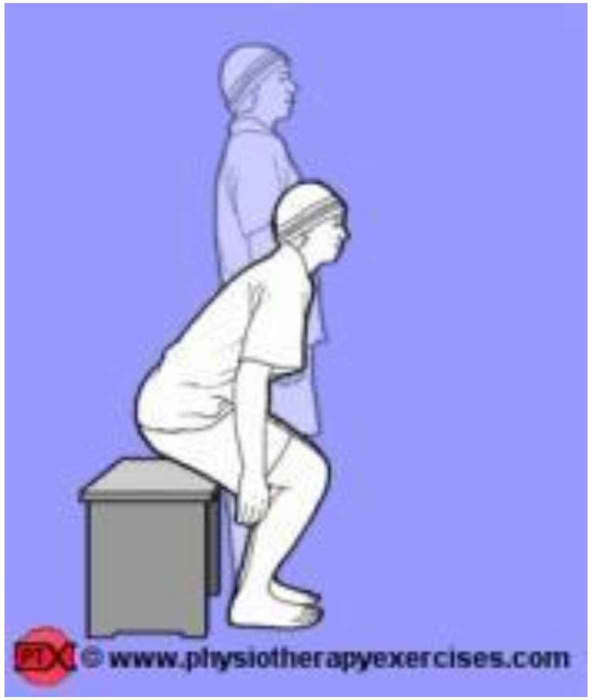

	Visit No:	Phone Call:	Phone Call:
How long:			
How many:			
How often:			
How fast:			
How much support:			

Comments: ____

____

Try to work at a moderate level of exertion without getting short of breath or fatigued.

IMPORTANT:

- Ensure the area around you is clear of unwanted objects
- Always have bare feet or shoes that fit firmly

STOP if you are in pain!

ACTIV
